# Facts, Dogmas, and Unknowns About Mitochondrial Reactive Oxygen Species in Cancer

**DOI:** 10.3390/antiox13121563

**Published:** 2024-12-19

**Authors:** Milagros Junco, Clara Ventura, Florencia Ximena Santiago Valtierra, Eduardo Nestor Maldonado

**Affiliations:** 1 Department of Drug Discovery & Biomedical Sciences, Medical University of South Carolina, Charleston, SC 29425, USA; mjunco@vet.unicen.edu.ar; 2 Virology Laboratory, Tandil Veterinary Research Center (CIVETAN), UNCPBA-CICPBA-CONICET, Tandil B7000, Argentina; 3 Institute for Immunological and Physiopathological Studies (IIFP), National Scientific and Technical Research Council (CONICET), Buenos Aires, La Plata 1900, Argentina; cventura@ffyb.uba.ar; 4 Department of Biology, Biochemistry and Pharmacy (DBByF), National University of the South (UNS), Bahia Blanca B8000, Argentina; fsantiagov@inibibb-conicet.gob.ar; 5 Hollings Cancer Center, Medical University of South Carolina, Charleston, SC 29425, USA

**Keywords:** antioxidants, cancer, lipid peroxidation, mitochondria, oxidative stress, ROS, VDAC

## Abstract

Cancer metabolism is sustained both by enhanced aerobic glycolysis, characteristic of the Warburg phenotype, and oxidative metabolism. Cell survival and proliferation depends on a dynamic equilibrium between mitochondrial function and glycolysis, which is heterogeneous between tumors and even within the same tumor. During oxidative phosphorylation, electrons from NADH and FADH_2_ originated in the tricarboxylic acid cycle flow through complexes of the electron transport chain. Single electron leaks at specific complexes of the electron transport chain generate reactive oxygen species (ROS). ROS are a concentration-dependent double-edged sword that plays multifaceted roles in cancer metabolism. ROS serve either as signaling molecules favoring cellular homeostasis and proliferation or damage DNA, protein and lipids, causing cell death. Several aspects of ROS biology still remain unsolved. Among the unknowns are the actual levels at which ROS become cytotoxic and if toxicity depends on specific ROS species or if it is caused by a cumulative effect of all of them. In this review, we describe mechanisms of mitochondrial ROS production, detoxification, ROS-induced cytotoxicity, and the use of antioxidants in cancer treatment. We also provide updated information about critical questions on the biology of ROS on cancer metabolism and discuss dogmas that lack adequate experimental demonstration. Overall, this review brings a comprehensive perspective of ROS as drivers of cancer progression, inducers of cell death, and the potential use of antioxidants as anticancer therapy.

## 1. Introduction

The capability of mitochondria to produce ATP and sustain oxidative metabolism was recognized in the early 20th century after the identification of oxidation reactions in the mitochondrial matrix. The chemiosmotic hypothesis of Mitchell linked oxidation of substrates with ATP synthesis. Mitchell postulated a proton (H^+^) electrochemical gradient across the inner mitochondrial membrane (IMM) as the energy-rich intermediate of oxidative phosphorylation (OxPhos) [1,2]. He proposed that electrons (e^−^) flow through complexes of the electron transport chain (ETC), inducing the translocation of H^+^ to the mitochondrial intermembrane space. H^+^ accumulation creates a proton motive force (Δp) used by the ATP synthase to synthesize ATP from ADP and inorganic phosphate. Studies on the e^−^ flow through the ETC have been key for the discovery of mitochondrial production of reactive oxygen species (ROS) caused by single e^−^ leaks. Beyond bioenergetics, mitochondria are a biosynthetic hub and participate in cell signaling. Overall, mitochondrial metabolism yields ROS, ATP, and metabolic byproducts used in the synthesis of amino acids, fatty acids, nucleotides, cholesterol, glucose, and heme.

Regardless of a relatively long history of research on cancer metabolism, mitochondrial metabolism and ROS in tumors only recently became a hot topic. A 2024 updated PubMed search using the words mitochondrial metabolism, cancer, and ROS showed 9426 total publications between 1990 and 2023, with only 384 (~4.1% of the total) between 1990 and 2005 and 4970 (~52.8% of the total) since 2018. Another search using the words ROS and cancer showed 37,262 publications, with 1981 publications between 1990 and 2005 (~5.3% of the total) and 22,086 (~59.2%) since 2018.

Accumulation of ROS in cancer cells has been referred to as a concentration-dependent double-edged sword because they serve as signaling molecules contributing to cellular homeostasis and proliferation or cause cell death. Regardless of the abundant research published on this matter, experimental data are still lacking to explain or determine several critical aspects. Which are the actual ROS toxic levels in primary and cancer cells? Is the rate of proliferation associated with ROS toxicity? Are specific ROS determining toxicity, or it is the cumulative effect of all of them? Are ROS-induced damages to DNA, protein, and lipids equally relevant to alter cellular homeostasis? Do they occur at the same time or are they sequential? Are ROS toxic levels specific for each cancer type and different between primary and metastatic tumors? Can ROS be manipulated to prevent proliferation without killing tumor cells?

In this review, we aim to provide an updated and critical perspective of the role and effects of mitochondrial ROS in cancer metabolism. We provide a comprehensive integral description combining older and newer basic concepts of ROS formation, deleterious effects, and cellular antioxidant systems. We also discuss dogmas that lack adequate experimental demonstration and how antioxidants could be used as adjuvants for cancer therapy. Overall, we describe mitochondrial ROS biology from a mechanistic perspective in relation to cancer cell proliferation, survival, and death.

## 2. Reactive Oxygen Species

### 2.1. Molecular Species

Reactive species are named based on the reactive atoms, which are oxygen (O_2_), nitrogen, or sulfur. Superoxide anions (O_2_•^−^), hydroxyl (HO•), peroxyl (ROO•), and alkoxy (RO•) radicals and hydrogen peroxide (H_2_O_2_) are formed by reduction-oxidation (redox) reactions derived from molecular O_2_. The distinct chemical reactivity of ROS spans up to 11 orders of magnitude in the second-order rate constants with specific targets [3,4]. H_2_O_2_ is the most important ROS in cell signaling and redox regulation [5]. Low intracellular concentrations of H_2_O_2_ (~1–100 nM) reported in hepatocytes [6] results from the balance between production and removal by reducing systems. By contrast, O_2_•^−^ is found at much lower concentrations (10^−11^ M) vs. (10^−8^ M) of H_2_O_2_ [7]. The maintenance of low-level H_2_O_2_ and its associated physiological redox signaling has been called oxidative eustress [6]. Superoxide dismutases (SODs) and catalase (CAT) detoxify O_2_•^−^ and H_2_O_2_, respectively. The highly toxic HO• and peroxynitrite (ONOO^−^), for which there are not specific detoxifying systems, do not regulate any biochemical pathway. Peroxyl and alkoxy radicals induce lipid peroxidation.

Precise information about physiological non-toxic ROS levels is very scarce. The homeostatic set point for ROS seems to be cell-type dependent. In general, concentrations around 10–100 nM are associated with cellular homeostasis and physiological cellular responses, while concentrations between 1–10 µM induce cell damage [6]. It has been estimated that ROS at ~7 µM induce apoptosis in Jurkat cells derived from acute lymphoid leukemia [8]. However, in normal rat thymocytes, steady-state concentrations of 42 and 83 nM were reported in control and apoptotic cells, respectively [9]. It has been proposed that cancer cells may release H_2_O_2_ at a lower rate than primary and immortalized cells, possibly due to higher antioxidant defenses [10].

### 2.2. Formation

#### 2.2.1. Mitochondria

The ETC and NADPH oxidases (NOX) are the major sources of O_2_•^−^ and H_2_O_2_. NOX and ETC in resting myoblasts produce ~40% and 45% of net cellular H_2_O_2_, respectively [11]. The flow of e^−^ through complexes I to IV of the ETC generates ROS by redox reactions in at least 11 different sites [12]. Generation of ROS in mitochondria is highly dynamic and influenced by fluctuations in mitochondrial metabolism.

ROS formation in the respiratory chain.

**Figure 1:**
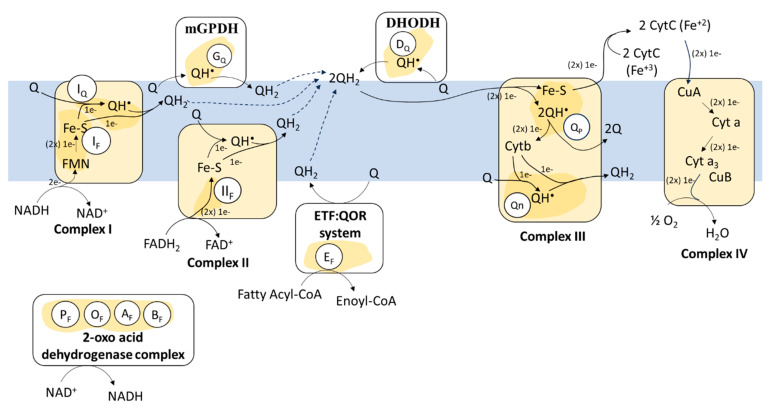
Mitochondrial sites of ROS production. Schematics of the electron flow in the electron transport chain showing the major sites of ROS production: IQ, IF, IIF, and QP. FMN: flavin mononucleotides cofactor; Fe-S: iron-sulfur clusters; mGPDH: mitochondrial glycerol-3-phosphate dehydrogenase; Q: ubiquinone; QH_2_: ubiquinol; ETF:QOR: electron-transferring flavoprotein-ubiquinone oxidoreductase; DHODH: dihydroorotate dehydrogenase.

Complex I: Redox reactions in the ETC start with the flow of 2 e^−^ from NADH that reduce flavin mononucleotides cofactor (FMN) at complex I. FMNH_2_ remains partially oxidized (FMNH^•^) after the transfer of one e^−^ to iron–sulfur clusters (Fe-S). Electrons from Fe-S clusters transferred to ubiquinone (Q), also known as Coenzyme Q, generate semiquinone (QH^•^), which in turn becomes ubiquinol (QH_2_) after accepting a second e^−^ [13]. At complex I, ROS are produced by e^−^ leaks at site I_F_ during the transfer between the FMN and the Fe-S groups and at site I_Q_, from the Fe-S centers to the ubiquinone [12,14,15,16] (Figure 1). In several cell lines, site I_Q_ produces up to two-thirds of the mitochondrial O_2_•^−^ and H_2_O_2_ [17].

Complex II: At complex II, e^−^ from succinate to FADH_2_ are transported to ubiquinone through a series of Fe-S centers in one e^−^ transfer reactions. ROS production at site II_F_ [12,18] has often been considered negligible because of the difficult access of O_2_ to the e^−^ leakage site that is occupied by dicarboxylic acids [19,20]. However, accumulating evidence suggest that complex II is a source and modulator of ROS production [18,21,22].

Complex III: At complex III, reduced coenzyme Q (QH_2_) transfers e^−^ to two molecules of cytochrome c (cyt c) in the mitochondrial intermembrane space through the Q cycle (Figure 1). QH_2_ enters the Qp site of complex III and transfers one e^−^ to a Fe-S cluster in the Rieske center and later to a cyt c as Fe^3+^, which accepts one e^−^ and is reduced to Fe^2+^. A second e^−^ is transferred to cytochrome b and then to a Q molecule which occupies the Qn site of complex III and is partially reduced to QH^•^. When a new QH_2_ molecule enters complex III, it undergoes the same set of reactions, transferring one e^−^ to a second molecule of cyt c and another to the Qn site, completing the reduction of QH^•^ forming QH_2_. Electron leakage from the QH^•^ to O_2_ mostly occurs at the Qp site, generating O_2_•^−^ [17,23,24,25].

Complex IV: At complex IV, four e^−^ are transferred almost simultaneously from fourmolecules of cyt c containing Fe^2+^ to one O_2_, generating two H_2_O. Each e^−^ that flows from cyt c to the binuclear copper center Cu_A_ is further transported to a heme group (cytochrome a) and later to another binucleate center comprising a heme-bound iron (cytochrome a3) and the nearby copper center (Cu_B_), where O_2_ reduction occurs without ROS production [20,26,27,28].

*Retro formation of ROS in the respiratory chain*. Reverse electron transport (RET) describes the e^−^ flow against a redox potential gradient within the ETC. High mitochondrial membrane potential (ΔΨ_m_) and high QH_2_/Q ratios, usually generated from complex II, cause RET [29]. Although most e^−^ flow from succinate to O_2_ increasing ΔΨ_m_ and the QH_2_ pool, some move against its redox potential from QH_2_ to NAD^+^. In addition to the QH_2_/Q ratio, pH, NADH/NAD^+^ ratios, and O_2_ also influence ROS production by RET at complex I [30]. RET modulation has been associated with cell differentiation [31], O_2_ sensing by arterial chemoreceptors [32], and modulation of the e^−^ flow in ETC. Moreover, the flow of e^−^ by RET alters NADH/NAD^+^ influencing the activity of NAD^+^-dependent sirtuins, and the Foxo transcription factor pathway in aging and age-related pathologies [33]. RET has also been related to the induction of apoptosis in cancer cells [34].

*Other mitochondrial sites of ROS formation***.** Several mitochondrial enzymes generate ROS. A fraction of ROS traditionally associated with complex I is in fact generated by dehydrogenases in the mitochondrial matrix [35]. In particular, 2-oxoglutarate dehydrogenase (site O_F_) and pyruvate dehydrogenase (site P_F_) contribute to mitochondrial ROS production in mouse liver [35,36]. In addition, mitochondrial glycerol-3-phosphate dehydrogenase (site G_Q_), the e^−^ transferring flavoprotein/ubiquinone oxidoreductase system (site E_F_), and dihydroorotate dehydrogenase (site D_Q_), which donate e^−^ to the Q system, also contribute to the production of mitochondrial ROS [12] (Figure 1). Mitochondrial cytochrome P450a that metabolize a wide range of endogenous substances and xenobiotics generate O_2_•^−^ and H_2_O_2_ as byproducts [37,38,39].

*ROS release*. At complexes I, II, and III, ROS are released to the matrix, but only from complex III do ROS reach the intermembrane space [11,40,41]

#### 2.2.2. ROS-Induced ROS Release (RIRR)

Sustained high ROS production can exceed the cellular antioxidant capacity, triggering a phenomenon known as ROS-induced ROS release (RIRR). During RIRR, high ROS induce the onset of the mitochondrial permeability transition pore (mPTP), which permeabilize mitochondrial membranes, causing loss of ions and solutes, mitochondrial dysfunction, and cell death [42]. After mPTP, ROS released to the intracellular space induces ROS formation in neighboring mitochondria [43]. RIRR generates a temporally and spatially coordinated response that allows signaling and increased ROS production. It has been proposed that an undefined not too high ROS level could trigger a transient and lower conductance mPTP that would contribute to mitochondrial homeostasis, possibly by regulating intramitochondrial Ca^2+^ [44,45,46]. However, RIRR is not completely inhibited by the mPTP blocker cyclosporin A, suggesting the existence of an internal mitochondrial membrane channel, which may function as alternative mPTP-independent permeation pathway [42,47].

#### 2.2.3. Non-Mitochondrial Sources

Enzymatic reactions in peroxisomes, endoplasmic reticulum, plasma membrane, and cytosol produce ROS. Acyl-CoA oxidase [48,49], urate oxidase [50], D-amino acid oxidase [51,52], polyamine oxidase [53], and xanthine oxidase [54] among others generate H_2_O_2_, O_2_•^−^ and nitric oxide (NO) in peroxisomes. Protein disulfide isomerase and ER oxyreductin-1 generate ROS within the endoplasmic reticulum. The major source of ROS production in the plasma membrane are the NOX family that are also found in mitochondria, endoplasmic reticulum, and perinuclear membranes [55,56,57]. It has been estimated that the endoplasmic reticulum can generate up to 25% of the total cellular ROS [58].

#### 2.2.4. Cancer Mutations

Mutations in complexes of the ETC in several types of cancers have been associated with abnormal ROS production and alterations of cell migration, invasion, and metastasis [59,60,61,62]. Table 1 shows gene mutations affecting ETC complexes, types of cancer holding the mutations, and effects on tumor progression.

## 3. Mitochondrial Antioxidant Systems

ROS are metabolized in mitochondria by enzymatic and non-enzymatic antioxidants [100,101,102,103].

### 3.1. Superoxide Dismutases (SODs)

SODs are the first line of defense against O_2_ free radicals. The dismutase activity of SODs accelerates the reaction of O2•^−^ with itself to form H_2_O_2_ and O_2_ (2 O_2_•^−^ + 2 H^+^ → H_2_O_2_ +O_2_). SOD-catalyzed dismutation is extremely efficient, occurring at the almost diffusion-limited rate of ∼2 × 10^9^ M^−1^ s^−1^, which is ∼10^4^ times the rate constant for spontaneous dismutation [104]. The three classes of SOD require different metal ion cofactors and have distinct subcellular localizations [105,106,107]. The Cu/Zn-SOD/SOD 1 is the cytoplasmic isoform, but it has also been identified in the nucleus, peroxisomes, lysosomes, and the mitochondrial intermembrane space [108]. Mn-SOD/SOD2 localizes to the mitochondrial matrix, the IMM, and cytosol [109]. The Cu/Zn-SOD/SOD3 is found in the extracellular space of tissues and extracellular fluids, including blood [108]. SOD1 is the most abundant in most tissues at a high concentration (10–40 µM). SOD 1 and SOD2 are expressed in almost every cell, whereas high expression of SOC3 has been identified in blood vessels, lung, kidney, and heart [110]. Loss of SOD activity is associated with increased oxidative damage, such as protein carbonylation, DNA breakage, and lipid peroxidation [111].

Antioxidant enzyme profiles change as tumors grow in several cancer types. Overexpression of SOD1 has been found in lung [112] and mammary tumors [113,114]. SOD1 is essential for the growth of non-small-cell lung cancer, leukemia, and other cancer cell lines [112,115,116]. Alterations in SOD2 gene expression and protein levels have also been found in different types of cancer cells [117]. SOD2 was overexpressed in prostate cancer compared to benign prostatic hyperplastic tissue [118], as well as in breast cancer [119]. Although it is not understood if SOD2 contributes to tumorigenesis, a possibility is that in aggressive tumor phenotypes, high expression of SOD2 could lead to increased mitochondrial production of H_2_O_2_ that activates oncogenic and angiogenic pathways [117]. Because of their potential role in tumorigenesis, SODs are considered promising anticancer drug targets [115].

### 3.2. Glutathione Peroxidase and Glutathione

GSH is a soluble tripeptide composed of glutamate, cysteine, and glycine present in high concentrations (1–10 mM) in the cytoplasm, nucleus, and mitochondria. Mitochondrial GSH (10% of total cellular GSH) is maintained by the flavoenzyme glutathione reductase, which reduces mitochondrial GSSH to GSH using NADPH [120,121,122]. GSH contains an active oxidizing and dehydrogenating thiol group that scavenges O_2_•^−^ and is an e^−^ donor for antioxidant enzymes such as glutathione peroxidase (GPX) [123]. GSH levels are high in ovarian, breast, and lung cancer and comparatively low in brain and liver tumors [124]. Although counterintuitive, high levels of GSH are associated with poor prognosis possibly because the increased ability to detoxify ROS increases the resistance to oxidative stress induced by chemotherapy [125,126]. GPX1 to GPX8 catalyze the reduction of H_2_O_2_ or organic hydroperoxides to H_2_O using reduced glutathione (GSH) as an e^−^ donor [127].

### 3.3. Catalase

CAT breaks down H_2_O_2_ into H_2_O and O_2_ [106]. The protective effect of CAT from oxidative injury induced by H_2_O_2_ and antimycin A has been demonstrated in HepG2 cells overexpressing mitochondrial CAT [128]. Several studies have shown that cells with higher CAT activity are more resistant to oxidative stress [129], while CAT inhibition sensitizes cells to H_2_O_2_ [130]. CAT inhibition by aminotriazole enhances the cytotoxicity of artesunate (inducer of ROS-dependent apoptosis) in HepG2 and A549 cells but not in HeLa cells, suggesting a cell-line specificity. CAT silencing leads to increased ROS-dependent cytotoxicity [131].

### 3.4. Thioredoxin

The thioredoxin system that comprises NADPH, thioredoxin reductase (TrxR), and thioredoxin (Trx) forms a critical disulfide reductase system for defense against oxidative stress [132,133]. Mammalian cells have a cytosolic Trx1 system and a mitochondrial Trx2 system [134,135]. The thioredoxin system provides e^−^ to thioredoxin-dependent peroxidases (Prx1 and 2), which can efficiently eliminate ROS. Additionally, it reduces methionine sulfoxide reductases and participates in protein repair. Trx regulates the activities of many oxidation-sensitive transcription factors, such as NF-κb [136], Nrf-2 [137], and p53 [138]. Trx-1 has been found to be overexpressed in prostate cancer [139], breast cancer [140], colon cancer [141], squamous cell carcinoma [142], and various types of leukemia [143].

### 3.5. Alpha Lipoic Acid (ALA)

ALA and its reduced form dihydrolipoic acid are antioxidants [144]. The antitumor effects of ALA have been associated with cell-cycle arrest at G1, increased levels of p53 [145,146], decreased proliferation, and downregulation of the anti-apoptotic proteins Mcl-1 and Bcl-xl in breast cancer cells [147,148]. ALA has also increased caspase-3 activity in breast cancer and neuroblastoma cell lines and decreased tumor size in animal models [149].

### 3.6. Coenzyme Q or Ubiquinone (Q)

Approximately 40% and 50% of Q is in the IMM, either as fully oxidized, partially reduced, or fully reduced [150]. In addition to its role as an e^−^ and H^+^ proton carrier for OxPhos, ubiquinol, which is the reduced form of ubiquinone, is an antioxidant [151,152]. Ubiquinol has also been shown to directly react with O_2_•^−^ [153]. Cancer cells lacking mitochondrial complex III have exhibited impaired tumor growth because ubiquinol oxidation by the ETC is essential for driving the tricarboxylic acid cycle [154]. Administration of supra-physiological concentrations of Q disrupts ETC function, leading to increased ROS and subsequent apoptosis in pancreatic cancer cell lines [155]. Q levels in breast tumor tissues have been found to be lower than in corresponding non-cancerous tissues, likely because it is consumed to prevent oxidative damage [156].

### 3.7. NADPH

NADPH is generated in the cytosol through the pentose phosphate pathway and in the mitochondrial matrix by isocitrate dehydrogenase 2 (IDH-2), glutamate dehydrogenase, malic enzyme (ME), and nicotinamide nucleotide transhydrogenase (TH). Deletion or downregulation of the IDH-2 gene, mutations in TH, or reduction of ME increase susceptibility to oxidative stress [157]. Cancer cells require high levels of NADPH for nucleotide synthesis through the production of S-phosphoribosyl-1-pyrophosphate (PRPP), the substrate for purine and pyrimidine synthesis [158,159]. NADPH is a cofactor of the glutathione reductase that forms GSH [160] and the reducing potential donor for most ROS detoxifying enzymes like thioredoxins, which in turn are utilized by glutaredoxins, peroxiredoxins, and glutathione peroxidases [161]. Thioredoxin reductase (TRXR) utilizes NADPH as an e^−^ donor to maintain the reduced form of TRX, thus contributing to the elimination of H_2_O_2_ [162]. NADPH also plays other metabolic roles, such as binding and reactivating CAT after it has been inactivated by H_2_O_2_ [163] and catalyzing the reduction of dihydrofolate (DHF) to tetrahydrofolate (THF) by the dihydrofolate reductase (DHFR) in the folate metabolism, ending in the synthesis of thymidylate, purines, methionine, and amino acids [164].

Mitochondrial antioxidant systems with the corresponding mechanisms of action are summarized in Table 2.

## 4. VDAC Links Mitochondrial Metabolism and ROS Formation

Voltage-dependent anion channels (VDAC) 1, 2, and 3 are β-barrel structures and the most abundant proteins in the outer mitochondrial membrane of eukaryotic cells [210,211,212,213]. The influx of oxidizable substrates, ADP, inorganic phosphate, and glycolytic ATP into mitochondria and the efflux of ATP through the outer mitochondrial membrane occurs only through VDAC [211,214,215]. Once inside the matrix, oxidizable substrates enter the tricarboxylic acid cycle, generating NADH and FADH_2_ that fuel the ETC. Overall, VDAC operates as a biological switch that maximizes the flux of metabolites for optimal mitochondrial function in the open state, whereas during the closed state, it lowers mitochondrial metabolism [216,217,218]. Thus, regulation of only this channel has an amplifying effect on several intra- and extra-mitochondrial pathways that modulate cancer metabolism and bioenergetics. Since mitochondrial ROS production depends mostly on the activity of the ETC, VDAC opening or closing is a major driver for ROS formation [216,219,220]. Moreover, VDAC regulation may serve as an adjustable rheostat with a range of operational levels that depends on the magnitude and duration of VDAC opening. Our group has demonstrated that free tubulin closes VDAC in cancer cells and dynamically modulates mitochondrial metabolism [221,222]. We have also identified small molecules that antagonize the inhibitory effect of free tubulin on the channel [219,222]. VDAC-tubulin antagonists work as VDAC openers, increasing oxidative metabolism and ROS formation. Treatment with VDAC openers caused oxidative stress and hepatocarcinoma cell death [220,223]. Recently, we have shown that the VDAC opener X1 has a synergistic effect with sorafenib, regorafenib, and lenvatinib, which are currently FDA-approved drugs for treating human hepatocarcinoma [224]. Overall, VDAC opening is an attractive mechanism to target pharmacologically and induce cytotoxic levels of ROS in cancer cells.

## 5. The Good and Bad ROS Levels

ROS signaling favors proliferation, contributes to the maintenance of homeostasis, and triggers beneficial stress responses [225]. However, excessive accumulation of ROS, commonly referred to as oxidative stress, damages DNA, lipids, and proteins [226]. Oxidative stress is a well-known cause of mitochondrial dysfunction, metabolic imbalances, and cell death linked to the progression of cancer, diabetes, and cardiovascular diseases [227,228].

### 5.1. The Triangle of Mystery and the Missing ROS Thresholds

**Figure 2:**
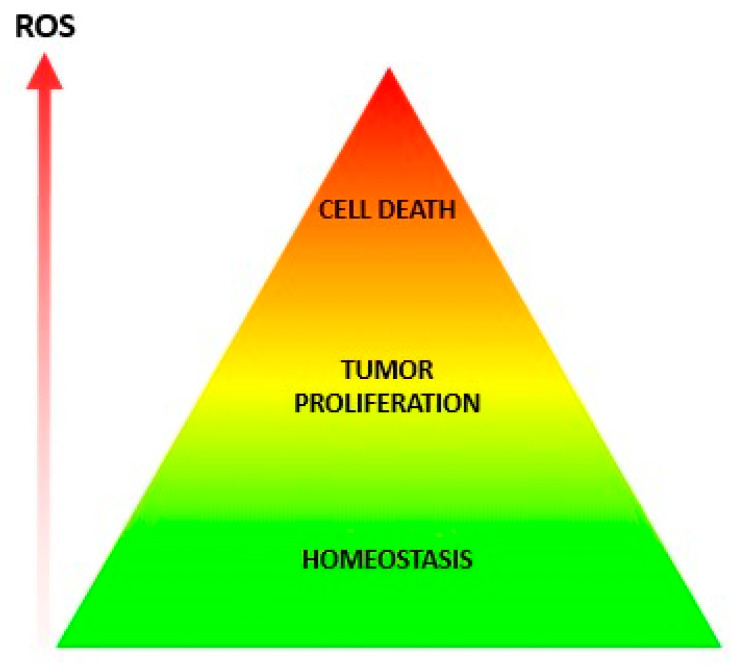
Impact of ROS levels on cellular function. At low levels of ROS, cells maintain homeostasis (green). Intermediate levels of ROS stimulate cell proliferation (yellow). High levels of ROS cause cell death (red). The continuous gradient illustrates the lack of defined levels separating each zone.

Several publications represent ROS concentrations in physiological and pathological redox states as a triangle divided into three different colored sections (Figure 2). The section corresponding to lower levels of ROS is described as physiological signaling that favors homeostasis. An intermediate section corresponds to moderate ROS levels that favor tumor-cell proliferation and invasion. A third section represents high ROS causing oxidative stress leading to oxidative damage and cell arrest or cell death. This triangle, conceptually intuitive and widely found in the redox literature, is not supported by sufficient experimental data, except for levels of H_2_O_2_ that have been calculated in specific systems like rat liver and isolated hepatocytes [4]. Several questions about this theoretical approach remain unanswered. What are the actual levels of ROS considered low, moderate, or high in different cells? Is there a general threshold for all ROS, or is there a threshold for each type? What is the level of each ROS species? Is this true for cancer and non-cancer cells as well? If so, to which type of cancer does the concept apply? Answers to these and other related questions may contribute to shedding light and adding details to the widely used conceptual approach to ROS levels.

### 5.2. ROS as Signaling Molecules

H_2_O_2_ is a versatile pleiotropic signaling agent and the major contributing ROS to cellular redox regulation [106,229,230]. It is relatively stable and can cross cellular membranes [5,231,232]. A steady-state physiological flux of H_2_O_2_ in the low nanomolar range (~1–100 nM) to specific protein targets leads to reversible oxidation of proteins. The major mechanism by which H_2_O_2_ attains specificity to mediate biological signaling effects is through oxidation of protein sulfur (thiolate groups) [233]. Redox signaling can also occur through reversible methionine oxidation [234], through selenoproteins [235], through oxidation of protein metal centers [236], and through oxidized lipids [237]. If the H_2_O_2_ concentration reaches supraphysiological levels (roughly estimated to be above 100 nM), unspecific oxidation of proteins, altered response patterns, and damage to macromolecules cause growth arrest and cell death [225].

ROS signaling modulates phosphatases, kinases, proteases, and transcription factors that influence cell proliferation and differentiation, as well as responses to hypoxia, aging, immunomodulation, circadian rhythms, aging, inflammation, autophagy, and cell survival [230,238]. H_2_O_2_ in the low micromolar range activates the canonical insulin signaling pathway dependent on IRS-1/PI3K/Akt, as well as other signaling like adenosine monophosphate-activated protein kinase (AMPK) and p38 MAPK, to increase basal glucose transport [239]. The transcription factor NF-κb plays an important role in coordinating innate and adaptive immunity, cell proliferation, apoptosis, and development. ROS influence the activation of the NF-κb pathway by inhibiting the phosphorylation of IκBα at the Tyr42 residue or at Cys179 of IKKβ, regulating the activation of MEEK1 [240]. Most studies on NF-κb activation have used H_2_O_2_ [241]. ROS also activate EGF receptors, stimulating RAS and subsequently activating the ERK pathway [242]. The phosphoinositide 3-kinase (PI3K)–Akt pathway is directly activated by ROS, leading to the formation of phosphatidylinositol 3,4,5-trisphosphate (PIP3), an important second messenger in cellular signaling [243]. Furthermore, ROS inactivate PTEN (phosphatase and tensin homolog), which inhibits Akt activation and allows its phosphorylation by casein kinase II (Ck2), further reducing its inhibitory activity on Akt. Additionally, ROS can deactivate protein phosphatase 2A (PP2A), which leads to Akt activation [244].

## 6. ROS Accumulation and Cytotoxicity in Cancer

### 6.1. Effects on DNA

DNA damage refers to physical or chemical changes in the DNA [245]. Unrepaired DNA damage activates cell-cycle control mechanisms halting proliferation. In cancer, uncontrolled proliferation is facilitated by a lower efficiency of DNA repair and suppression of cell-cycle checkpoints [246,247]. ROS oxidize nucleobases. Guanine (G), with a lower oxidation potential compared to other bases, forms the oxidized product, 7,8-dihydro-8-oxo-2′deoxyguanosine (8-oxo-dG) [248]. This modification induces mutagenesis due to loss of base-pairing specificity and mispairing with adenine (A). When ROS oxidize G in DNA, 8-oxo-dG acts as a template, resulting in G-T (thymine) transversion mutations. When ROS oxidize deoxyguanosine triphosphate in the nucleotide pool, 8-oxo-dG acts as a substrate, resulting in A-C (cytosine) mutations. ROS also oxidize A with the production of intermediates that cause A-C, A-G, A-T substitutions, and the GC-AT transition [249]. Furthermore, ROS can break hydrogen bonds, unfolding the DNA double-helix structure, leading to the exposure of more purine and pyrimidine residues to ROS, facilitating further nucleobase oxidation and additional DNA mutations.

The tumor suppressor gene p53 is a redox-sensitive transcription factor that orchestrates a variety of DNA-damage-response mechanisms. Oxidation is one of the causes of p53 mutations, which are found in 50–60% of human cancers [250,251,252,253]. ROS oxidize cysteines in p53 that affect transcriptional activity [254]. Mutations in p53 result in the loss of wild-type function favoring angiogenesis, metastasis, therapy resistance, and inactivation of other p53 family members also involved in tumor suppression [255,256,257].

### 6.2. Effect on Proteins

ROS oxidize amino acid residues, cleave peptide bonds, and aggregate proteins [258]. Oxidized proteins are hydrolyzed or processed by proteasomes. In general, ROS interact with specific amino acid residues that activate downstream kinase cascades, acting as switches for subsequent protein functions [259].

The mammalian mitogen-activated protein kinases (MAPKs), including c-Jun NH2-terminal kinase (JNK), p38 MAPK, and extracellular signal-regulated kinase (ERK), are serine-threonine protein kinases that regulate proliferation, differentiation, apoptosis, survival, inflammation, and innate immunity [260]. ROS activate MAPK pathways by oxidative modification of intracellular kinases such as ASK-1. Oxidized thioredoxin dissociates from ASK-1, leading to JNK and p38 pathway activation via ASK-1 oligomerization. Another potential mechanism for MAPK activation by ROS involves the inactivation and degradation of MKPs that maintain the pathway inactive. ROS-induced activation of MAPK pathways is blocked by antioxidants [261].

The low molecular weight RAS superfamily of proteins is activated by binding to guanosine triphosphate (GTP) and inactivated by guanosine diphosphate (GDP). ROS stimulate dissociation of guanine nucleotides from RAS in vitro and positively regulate RAS function in vivo [262]. GTP binds and activates the three RAF family proteins, RAF1, BRAF, and ARAF. Activated RAF phosphorylates mitogen-activated protein kinases/extracellular signal-regulated kinase 1 and 2 (MEK1 and MEK2), which activates the mitogen-activated protein kinases ERK1 and ERK2 [263]. ERKs’ phosphorylate transcription factors, such as ELK1 and c-Jun, lead to cell proliferation. RAS mutations and overexpression lead to uncontrolled cell proliferation [264]. RAS also activate PI3K that phosphorylates PIP2 to produce PIP3, which in turn activates Bruton’s tyrosine kinase (BTK) and AKT protein kinases. The proliferative and anti-apoptotic actions of AKT are crucial steps in the development of colon, pancreatic, breast, lung, and prostate cancers [264,265,266].

### 6.3. Mitophagy

Oxidative damage turns mitochondrial dysfunctional. Accumulation of dysfunctional mitochondria has been associated with tumorigenesis [267,268,269]. Mitophagy is the process by which autophagosomes selectively target mitochondria for degradation [270]. The regulatory mechanisms of mitophagy mediated by ROS include the Pink1/Parkin [271], HIF-BNIP3/Nix [272], and FOXO3-LC3/BNIP3 [273] signaling pathways. Depolarized mitochondria recruit Parkin to the outer mitochondrial membrane for further transport to lysosomes [274]. Additionally, Parkin induces the degradation of Mitofusin 1 (MFN1) and MFN2, which are essential for mitochondrial membrane fusion, thus promoting mitochondrial fragmentation and subsequent autophagic engulfment [275]. BNIP3 is a HIF-1α target induced by hypoxia, which promotes mitophagy, reduces mitochondrial mass, decreases overall oxygen consumption, and promote survival under hypoxic conditions [276]. Moreover, the loss of BNIP3 in a murine model of mammary tumorigenesis reduced mitophagy and increased mitochondrial ROS levels, resulting in increased normoxic stabilization of HIF-1α, increased Warburg effect, and enhanced tumor progression [277]. Oxidative stress also activates the forkhead box O3 (FOXO3) transcription factor, stimulating the transcription of LC3 and BNIP3, which are involved in autophagy-lysosome systems [278].

Although proteins involved in mitophagy are dysregulated in cancer patients, it is a matter of debate if they promote or suppress tumor growth, which seems to largely depend on the cancer type [279]. PINK1 and BNIP3 are both upregulated in lung cancer [280,281], while in ovarian cancer, PINK1 is downregulated, and BNIP3 is upregulated [282]. The NIX pathway is upregulated in breast cancer and downregulated in prostate cancer [283]. Within the non-canonical pathways, BCL2L13 is upregulated in leukemia [284] and downregulated in breast cancer [285].

### 6.4. Effects on Lipids, Lipid Peroxidation, and Mitochondrial Dysfunction

Alteration of the mitochondrial lipidome and lipid peroxidation induced by oxidative stress is currently considered a major cause of mitochondrial dysfunction in cancer, diabetes, cardiomyopathy, and neurodegenerative diseases [286,287,288]. Mechanistically, oxidative modifications of the mitochondrial lipidome alter cellular bioenergetics and metabolism through changes in membrane fluidity, lipid–lipid and lipid–protein interactions, activity of the respiratory supercomplexes, and modifications in the flux of ions and metabolites [289,290,291].

#### 6.4.1. Mitochondrial Lipid Composition and Function

Lipids influence the structure and fluidity of mitochondrial membranes [292,293], cristae morphology [294,295], mitochondrial fission and fusion [296,297,298,299], protein biogenesis and translocation [300,301], assembly of proteins of the ETC in supercomplexes [302,303], and apoptosis [304,305].

The outer mitochondrial membrane (OMM) is a lipid-rich surface, whereas the highly folded inner membrane has a higher protein–lipid content. Phosphatidylcholine (PC) and phosphatidylethanolamine (PE) comprise ~80% of total phospholipids (PL) in both membranes, whereas phosphatidylserine, phosphatidylinositol, and phosphatidic acid are in low proportions. The IMM is also enriched in cardiolipin (CL), found almost exclusively in mitochondria (Table 3) [306]. Ceramides and sphingomyelin together with gangliosides are other minor mitochondrial lipids extensively investigated for their roles in cellular metabolism [307,308]. On a relative basis, the OMM contains more cholesterol than the IMM, although the content is much lower compared to the plasma membrane (2–4% vs. 25–30% of the total cellular cholesterol, respectively) [309]. Overall, the content of cholesterol and sphingolipids is lower in mitochondria compared to other cellular membranes. Noticeably, mitochondrial cholesterol is higher in cancer than in primary cells, like is the case of human, rat, and mouse hepatocellular carcinoma (HCC) [310]. It has been proposed that accumulation of mitochondrial cholesterol in HCC contributes to chemotherapy resistance by reducing mitochondrial membrane permeabilization, avoiding the release of pro-apoptotic factors, and preventing cell death [309,310]. Mitochondrial lipid composition varies among cell types and dynamically adapts to changes occurring both in physiological and pathological conditions. A better understanding of alterations in mitochondrial lipid content and lipid molecular species in each cancer type might contribute to develop novel lipidomic-based cancer diagnosis and therapeutics [311,312,313,314,315,316,317].

#### 6.4.2. Lipids During Cell Division

PL, sphingolipids, cholesterol, cholesterol esters, and fatty acids (FA) form new membranes synthesized before cell division. In cancer cells, ROS either induce lipid accumulation or promote FA acid oxidation. Under different prooxidant conditions, ROS block lipolysis and cause lipid accumulation [318]. In liver cancer cell lines under hypoxia, HIF-1α suppresses the expression of long-chain acyl-CoA and medium-chain acyl-CoA dehydrogenases leading to decreased FA oxidation and ROS formation [319]. In HepG2 hepatocarcinoma cells, treatment with H_2_O_2_ induces overexpression of perilipin-2 (PLIN-2), resulting in increased content of lipid droplets (LDs) [320]. Overexpression of mitochondrial elongation factor 2 (MEF2) drives ovarian cancer progression by enhancing lipid accumulation. In this system, MEF2 induces ROS-mediated activation of the AKT/mTOR signaling pathway, resulting in increased LDs. By contrast, the cellular energy sensor AMPK, which is activated by ROS, induces lipolysis and FA oxidation. AMPK regulates FA metabolism not only by activating FA oxidation but also by inhibiting the fatty acid synthase through phosphorylation of acetyl-CoA carboxylase 1 and 2 (ACC1 and ACC2), which would otherwise block CPT-1 by producing malonyl-CoA. This regulation promotes the survival of tumor cells during stress conditions by maintaining ATP homeostasis and providing NADPH [321,322].

#### 6.4.3. Lipid Peroxidation

Lipid peroxidation, associated with the pathogenesis of cancer, [323,324,325], diabetes [326], atherosclerosis, and neurodegenerative diseases [327], is the process by which free radicals oxidize lipids containing carbon–carbon double bonds. In cellular membranes, polyunsaturated fatty acids (PUFAs) with two or more double bonds, esterified on PLs and free cholesterol, are the lipids most susceptible to peroxidation [328]. Linoleic acid (LA, 18:2n-6), arachidonic acid (AA, 20:4n-6), eicosapentaenoic acid (EPA, 20:5n-3), and docosahexaenoic acid (DHA, 22:6n-3) are the major PUFAs. The reactivity of PUFAs with free radicals increases with the number of double bonds [329].

**Figure 3:**
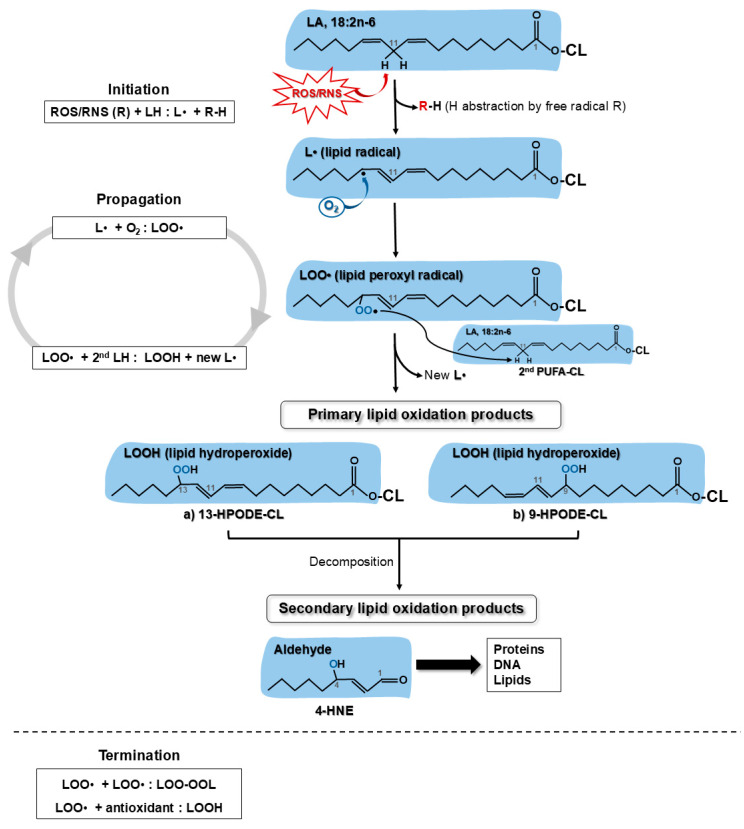
Peroxidation of linoleic acid (LA) in cardiolipin (CL) induced by ROS/RNS. General steps and reactions of chain lipid (L) peroxidation process initiated by a free radical (R) (left). Representation of the peroxidation cycle of one linoleic acid esterified into mitochondrial CL (right). PUFA hydrogen atoms at bis-allylic position (C11 in LA) are attacked by free radicals at initiation, or by lipid peroxyls during propagation. Lipid hydroperoxides, such as 13-HPODE-CL (a) or 9-HPODE-CL (b), are the primary oxidation products of CL, which further undergo decomposition, generating the secondary oxidation product, aldehyde 4-HNE. This highly reactive truncated lipid forms covalent adducts with cellular and mitochondrial DNA, proteins, and lipids.

Oxidation of lipids occurs through a chain reaction of initiation, propagation, and termination [330], as shown in Figure 3. Initial oxidation of PUFAs by HO•, O_2_•^−^, or ROO•^−^, as well as by reactive nitrogen species (RNS), like NO, ONOO^−^ or nitrogen dioxide (NO_2_•), originates a highly unstable lipid radical (L•). Lipid radicals are also formed by metal ions, ionizing radiation, UV light, heat, smoking, air pollution, or via enzymatic oxidation catalyzed by lipoxygenases [331] and cyclooxygenases [332]. During propagation, L• reacts with O_2_, forming the very unstable lipid peroxyl radical (LOO•) that reacts with other PUFAs generating another L• and a lipid hydroperoxide (LOOH). The newly generated L• perpetuates a self-propagated deleterious peroxidation cycle of free radicals producing new reactive radicals. Termination occurs when two lipidic free radicals react and produce a non-radical and non-propagating product. Many antioxidants, such as tocopherols, flavonoids, polyphenols, and vitamin E, facilitate termination of radical chain oxidation by donating a H^+^ to the LOO• species, resulting in the formation of stable LOOH non-radical products [333]. LOOH, are further decomposed into volatile (hydrocarbons, aldehydes, alcohols, and ketones) and non-volatile (aldehydes, oxidized triacylglycerols and their polymers) secondary non-radical lipid oxidation products [333]. Among these, the most extensively studied are the aldehydes malondialdehyde (MDA) and 4-hydroxynonenal (4-HNE) [334]. LOOH and the secondary aldehydes MDA and 4-HNE are widely recognized as biomarkers of lipid peroxidation and oxidative stress [335,336,337,338,339]. The formation of CL-derived lipidic peroxidation products is briefly schematized in Figure 3. The complexity of the chemical reactions involved in lipid peroxidation have been well described in detail [328].

#### 6.4.4. Alteration of Mitochondrial Function Induced by Oxidized Cardiolipin and Phosphatidylethanolamine

**Figure 4:**
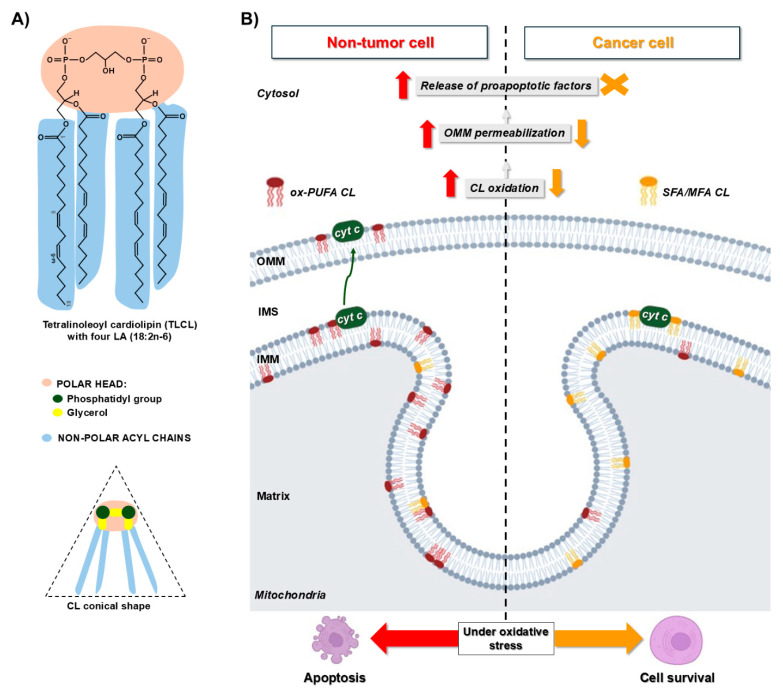
Reduction of CL oxidation confers resistance to apoptosis in cancer cells. (**A**) Chemical structure of tetralinoleoyl cardiolipin (TLCL), the most common CL molecular species in mammalian mitochondria. This non-bilayer phospholipid acquires a conical shape due to its four hydrophobic polyunsaturated acyl chains and directly binds and anchors proteins on the IMM, including apoptotic cytochrome c (*cyt c*). (**B**) Under oxidative stress, increased peroxidase activity exerted by *cyt c* forms oxidated cardiolipin (ox-CL), which easily flips from IMM to OMM and binds to Bid, promoting membrane permeabilization and release of mitochondrial pro-apoptotic factors. In addition, ox-CL has a looser interaction with *cyt c*, allowing its liberation to the mitochondrial intermembrane space (IMS) and then to the cytosol. Ox-CL is less efficient in stabilization of respiratory supercomplexes, leading to increased electron leakage and ROS generation. Molecular species of CL with saturated (SFA) and monounsaturated (MFA) fatty acids are enriched in cancer cells. Reduction of CL PUFA content and oxidation contribute to avoiding apoptosis in an oxidative environment, leading to tumor cell survival and chemoresistance.

CL and PE are major targets for oxidation due to the high proportion of PUFAs and the proximity to the ROS-producing ETC proteins [340]. CL is a PL with three glycerol backbones and four PUFAs (Figure 4A), with LA being the most common in animal tissues, comprising up to 80–90% in mammalian heart CL [341,342,343,344,345]. CL interacts with different IMM proteins, including complexes of the ETC, the ATP synthase [346,347,348,349], and the protein translocase complex TIM23 (translocase of the inner mitochondrial membrane 23) [350]. The role of CL in the assembly, stabilization, and function of the ETC supercomplexes has been extensively studied [302,349,351,352,353,354]. In CL-deficient mitochondria, the e^−^ flow through the ETC is disrupted, and the synthesis of ATP and ΔΨm are reduced [355,356,357,358]. Cytochrome c, located in the intermembrane space, forms a complex with CL and plays an important role during the early stages of mitochondrial-dependent apoptosis [359]. It has been reported that the complex cyt c–CL exerts peroxidase activity against CL PUFAs, resulting in the formation of oxidized CL hydroperoxides, such as CL-OOH and CL-OH, which permeabilize mitochondrial membranes favoring the release of pro-apoptotic factors to the cytosol [360,361]. Oxidative or nitrosative chemical modification [346,347,348,349] of *cyt c* increases its peroxidase activity, which is negligible in the non-oxidized form [360,362,363,364]. Although it seems paradoxical that mitochondria contain a unique lipid class so sensitive to oxidation, it could be speculated that CL is a sensor of oxidation working as an antioxidant in normal conditions or triggering apoptosis under oxidative stress.

The potential implications of mitochondrial lipid peroxidation on cancer cells’ resistance to apoptosis is poorly understood (Figure 4B). Oxidized and non-oxidized CL have been found to be lower in HCC than in adjacent non-cancerous tissues. Tumor CL contained less PUFA [365]. Furthermore, the HCC cell lines HepG2, Huh7, and LM3 stimulated with LA-rich CL-containing liposomes became more sensitive to apoptosis induced by sorafenib. Higher levels of oxidized CL have been found in mitochondria of apoptotic Huh7 cells stimulated with CL liposomes compared to non-stimulated controls. It has been proposed that a lower content of PUFAs in tumor cells decreases susceptibility to pro-apoptotic CL oxidation [365]. CL has been proposed to induce mitophagy. The externalization of CL to the OMM in damaged mitochondria act as a signal for autophagosome membrane-bound LC3 proteins. Removal of damaged mitochondria prevents CL oxidation and accumulation of pro-apoptotic signals [366]. Interestingly, overexpression of tyrosine aminotransferase (TAT) on gallbladder cancer promotes in vivo liver metastasis by potentiating CL-dependent mitophagy. Mechanistically, TAT directly binds to CL and leads to its exposure, inducing mitophagy [367]. Chemoresistance to paclitaxel-induced apoptosis of triple-negative breast cancer cells has been correlated with increased mitochondrial PC, PE, and CL [368]. Total CL was lower in mitochondria isolated from different types of mouse brain tumors compared to normal brain tissue. Molecular species forming CL may be unique for each tumor type [311]. For instance, chemically induced astrocytomas and ependymoblastomas from B6 mice exhibit a deficiency in mature CL molecular species with long-chain PUFAs and enrichment in immature CL species containing shorter saturated and monounsaturated acyl chains [311].

Information about the effects of oxidized PUFA-rich mitochondrial PE on mitochondrial function is relatively scarce. PE deficiency alters mitochondrial morphology and decreases respiratory capacity and ATP synthesis. Most of the studies on PE-deficient mitochondria have been conducted in yeasts. In mammalian CHO cells, a depletion of less than 30% of mitochondrial PE resulted in extensively fragmented and aberrant mitochondria with impaired formation of supercomplexes containing complexes I and IV without decreases in ΔΨm [303]. Similarly, loss of mitochondrial PE reduced respiratory supercomplexes’ formation in mouse muscle cells [369]. In contrast, depletion of mitochondrial PE in yeasts decreased ΔΨm due to cyt c impaired activity, caused by the formation of larger megacomplexes [370]. Contradicting those findings, other studies found that mitochondrial PE depletion only affected the activity of complexes III and IV but not the assembly of the supercomplexes [371]. Most recently, PE binding to the *cyt bc1* complex (III) was found to be important to maintain respiration in yeasts [372].

Due to the critical role of mitochondrial lipids in maintaining structure and activity of the ETC complexes, it is expected that modifications in the mitochondrial lipidome occurring in cancer cells have an impact on bioenergetics, ROS production, cell proliferation, and cell survival, as described above. Overall, CL and PE are essential to ensure the structure and function of the ETC and the ATP synthase. Peroxidation of both CL and PE alters mitochondrial bioenergetics in cancer and other diseases. However, CL peroxidation may also prevent the harmful effects of excessive ROS accumulation leading to apoptosis. Therefore, reduction of CL oxidation in cancer cells may be a mechanism to evade apoptosis. In any case, the biological consequences of oxidation or modifications of mitochondrial lipids are still incompletely understood.

#### 6.4.5. Aldehyde-Induced Damage to DNA and Proteins

A broad range of toxic products derived from oxidized lipids includes the reactive aldehydes MDA, 4-HNE, acrolein, crotonaldehyde, and glyoxal, which form covalent adducts with proteins and DNA **[373,374]**. Although it is generally accepted that ROS levels are high in most cancer types, the concentration of lipid oxidation products in tumors is still a matter of debate. Oxidation of mitochondrial CL generates 4-HNE, 13-hydroxyoctadecadienoic acid-CL (13-HODE-CL), and EAA-CL, all of them associated with decreased tumor progression [365].

Formation of 4-HNE has been demonstrated in vivo and in vitro [375,376]. Protein targets of 4-HNE include oxidoreductases, transferases, hydrolases, lyases, kinases, carriers, receptors, and ion channels. 4-HNE forms adducts with cysteine, histidine, and lysine which, in most cases, inhibit and few times increase protein activity [377,378]. In different cell types and subcellular fractions including liver mitochondria, 4-HNE binding to proteins represents up to ~8.5% of the total HNE adducts, 30% of which corresponds to mitochondrial proteins [379]. Among these are the trifunctional enzyme subunit α of the tricarboxylic acid cycle, the ATP synthase subunit β, the succinate dehydrogenase (ubiquinone) flavoprotein subunit, and the NADH dehydrogenase (ubiquinone) iron–sulfur protein 2. Binding to 4-HNE reduces mitochondrial respiration [380]. The effects of lipid peroxidation-derived aldehydes 4-HNE, MDA, and acrolein on mitochondrial proteins have been shown in heart, brain, kidney, muscle, and liver [381]. 4-HNE protein adducts accumulate both in nuclei and mitochondria from human kidney cancer compared to normal kidney tissue [382]. Other studies reported increased levels of MDA and 4-HNE in colorectal cancer tissues [383]. In thyroid tumors, 4-HNE content was higher than in normal tissue [384]. Formation of 4-HNE protein adducts has been related to the progression of kidney and colon cancers [385], while increased levels of 4-HNE have been associated with liver cancer initiation in animal models and humans [386]. Increased 4-HNE levels have also been found in invasive forms of breast carcinomas [387]. However, 4-HNE and related adducts are not always increased in tumors [388,389]. 4-HNE protein adducts have been found to be more concentrated in the non-malignant surrounding tissue than in human lung cancerous tissue, the difference being more evident in metastatic tumors compared to primary malignancies [390]. Similarly, in human colon adenocarcinoma [391] and HCC, [365] the concentration of 4-HNE was lower than the adjacent non-cancerous tissue. In addition, serum levels of MDA and 4-HNE are lower in gastric cancer patients compared to healthy subjects [392]. It could be speculated that aggressive cancer cells produce less reactive aldehydes due to a higher ratio of saturated and monounsaturated/polyunsaturated FA that limits peroxidation.

Aldehydes also form exocyclic adducts with DNA, introducing an extra ring on the nitrogen base of nucleotides, causing mutations or changes in the reading frame. Cumulating evidence links nuclear DNA alteration induced by lipid-derived aldehydes with carcinogenesis. The major adduct pyrimido[1,2α] purin-10 (3H) -one (M1G) formed between MDA and nuclear DNA, is highly mutagenic [393,394,395]. Increased levels of M1G have been found in human larynx [396] and breast [397] cancers. Levels of M1G in tobacco smokers are correlated with the silencing methylation levels of the hypermethylated cancer 1 tumor suppressor [398]. The 4-HNE-dG adduct, formed between 4-HNE and nuclear DNA [399], induces mutations of the tumor suppressor p53 gene [400,401]. The carcinogenic potential of 4-HNE may be related not only to DNA damage but also to the inhibition of DNA repair [402]. Another effect of 4-HNE is its capability to induce proliferation in normal cells. Physiological concentrations of 4-HNE increased cell proliferation in human B lymphocytes infected with Epstein–Barr virus [403] and in human hepatic stellate cells (hHSC) [404].

In addition to damaging nuclear DNA, ROS and reactive aldehydes are harmful to mitochondrial DNA (mtDNA), which is more susceptible to oxidative damage than nuclear DNA [405]. Oxidative modifications of mtDNA, like the formation of 8-oxo-dG, 4,6-diamino-5-formamidopyrimidine (FapyA), 2,6-diamino-4-hydroxy-5-formamidopyrimidine (FapyG), and thymine glycol, cause mutations, deletions, and strand breaks. Because mtDNA codifies only for 13 proteins of the respiratory chain, mtDNA mutations disturb the activity of the ETC. It has been proposed that mtDNA modifications caused by lipid peroxidation may be responsible for altered mitochondrial function in aggressive cancers [406,407]. High levels of 8-oxo-dG in mtDNA were found in patients with skin [408], urinary tract, bladder, and kidney cancer [409] and in the C6 glioma cell line [410]. Regardless, specific information about aldehydes or other lipid peroxidation products causing mtDNA alterations is still lacking.

#### 6.4.6. Lipid Peroxidation Products as Signaling Molecules

MDA and 4-HNE also are bioactive signaling lipids. In general, oxidized lipids have been implicated in signal transduction cascades during apoptosis, proliferation, inflammation, stimulation of adhesion molecules, cellular metabolism, and chemoattraction [334,411]. Enzymatic oxidation of AA by COX and LOX generates eicosanoids, including prostaglandins, thromboxanes, and leukotrienes, which play major regulatory roles in immune and inflammatory responses [412]. In addition, the prostaglandin-like isoprostanes produced by non-enzymatic oxidation of AA have been found to be increased in freshly isolated brain mitochondria subjected to Fe^2+^-mediated lipid peroxidation, suggesting a role for these molecules in mitochondria-mediated oxidative stress [413,414].

#### 6.4.7. Ferroptosis and Lipid Peroxidation

Ferroptosis is an iron-dependent form of nonapoptotic cell death characterized by excessive accumulation of iron, ROS-dependent lipid peroxidation, and alterations of mitochondrial morphology, including increased membrane density and reduction of mitochondrial cristae [415,416,417,418]. Iron is an essential cofactor of proteins of the ETC, including iron–sulfur (Fe-S)-cluster-containing proteins and heme-containing proteins [419]. However, the precise mechanism by which iron contributes to mitochondria-induced ferroptosis is unknown. A major cellular defense system against ferroptosis is the antioxidant system GPX4/GSH. GPX4 acts as an antioxidant enzyme reducing LOOH to less harmful lipid alcohols, even when those are integrated into membranes or lipoproteins [420,421]. Ferroptosis induced by depletion of intracellular GSH, the main reducing substrate for GPX4, together with decreased activity of this enzyme, leads to the accumulation of LOOH, which results in excessive ROS formation and irreversible oxidative damage to the mitochondria, bioenergetics failure, and cell death [422,423]. There is a consensus that lipid peroxidation has a central role in mitochondrial dysfunction during ferroptosis. Very recently, it has been shown that peroxidation of mitochondrial membrane lipids by ROS generated in the complex I of the ETC precedes the onset of ferroptosis [424].

## 7. Antioxidants as Adjuvants in Anticancer Therapies

Redox metabolism became a target to develop adjuvant therapies to treat cancer either by promoting the activity of antioxidant enzymes, inhibiting enzymes that increase ROS formation, or using exogenous antioxidants.

The nuclear factor erythroid (Nrf2) Kelch-like ECH-associated protein 1 (Keap1) system is an anti-stress mechanism that is dissociated by high levels of ROS. Free Nrf2 translocates to the nucleus and promotes the expression of antioxidant genes [425]. Current development of Nrf2 activators primarily focuses on modifying the sensor cysteines of KEAP1 to disrupt the KEAP1–NRF2 interaction [426,427]. Pharmacological agents targeting NOX could also become promising for cancer therapy [428]. The peptide Nox2ds-tat inhibits NOX2 assembly and the angiotensin II-induced production of O_2_•^−^. Additionally, various synthetic SOD mimetics with a rate of constants lower than constitutive SODS, have been effective in extracellular fluids where antioxidant enzymes are absent or in low concentrations [429,430]. The role of SOD3 has been investigated in numerous human and animal studies, revealing the harmful effects of reduced SOD3 levels and activity, as well as the beneficial effects of enhanced SOD3 in preventing oxidative damage [431,432].

Exogenous antioxidants like vitamins C and E, β-carotenes, resveratrol and other flavonoids, selenium, and N-acetylcysteine (NAC) are widely used as supplements. Because of the potential use as adjuvants in anticancer therapy, several antioxidants have entered clinical trials. In randomized trials, the antitumor efficacy of antioxidants has yielded mixed results, ranging from a protective effect to neutral or even promoters of some types of cancer. These apparently contradictory results, which are a matter of debate and intense research, may be caused, at least in part, by differences in the actual level of ROS during cancer cell proliferation and after chemotherapy. For example, vitamin C prevented DNA damage induced by H_2_O_2_ in lung cancer [433]. In patients with metastatic colon cancer, high doses of vitamin C did not prolong progression-free survival [434]. A phase 2 randomized trial is currently underway for patients with pancreatic cancer, and a trial focused on combination therapy with docetaxel in men with metastatic prostate cancer has recently concluded. This study revealed that intravenous administration of high doses of vitamin C showed no efficacy when combined with docetaxel [435]. Conversely, a study conducted by [436] indicated that prostate cancer patients with low selenium levels are more susceptible to DNA damage induced by ionizing radiation. Combination of selenite with docetaxel showed a synergistic effect in prostate cancer [437]. An update on active clinical trials involving antioxidants for cancer prevention and treatment can be found in a very recent review [438].

## 8. Concluding Remarks

ROS play a multifaceted role in cancer cell survival and proliferation. Cancer cells exhibit persistently high levels of ROS due to genetic, metabolic, and microenvironmental alterations. Relative ROS accumulation, which would be detrimental to normal cells, facilitates tumor growth. In cancer cells, ROS production and elimination systems are upregulated, keeping ROS levels below a still-undefined toxic threshold. Understanding the intricate interplay between ROS and cellular components may provide novel insights into the mechanisms driving cancer progression, likely leading to the identification of novel therapeutic targets for intervention strategies. Thus, modulating ROS levels and their downstream effects on cellular pathways could become a source of novel therapeutics in the future.

## Figures and Tables

**Table 1:** Mutations of the electron transport chain components in cancer.

ETC Complex	Gene	Cancer Type	Complex Activity	Effect on Tumor Progression	Reference
**Complex I**	MT-ND1, MT-DN2, MT-ND3, MT-ND4, MT-ND4L, MT-ND5, MT-ND6	Colon	↑ ROS production	↑ metastatic ability	[63,64,65,66,67]
	MT-ND2, MT-ND4, MT-ND5	Thyroid	↓ complex activity ↑ ROS production	↑ carcinogenesis	[68]
	ND1, ND2, ND3, ND4, ND4L, ND5, CytB, COX1, COX2, COX3	Pancreas	ND	ND	[69,70]
	MT-ND1, MT-ND2, MT-ND3, MT-ND4, MT-ND5	Breast	↓ complex activity	↑ metastatic potential ↑ cancer risk	[71,72,73]
	MT-ND1, MT-ND2, MT-ND4, MT-ND5	Renal	ND	↑ recurrence after surgery	[74,75]
	MT-ND3, MT-ND5, MT-ND6,	Lung	↓ complex activity ↑ ROS production	↑ metastatic ability ↑ proliferation	[64,76,77,78]
	MT-ND1, MT-ND2, MT-ND4L, MT-ND6	Leukemia	↓ complex activity	ND	[79,80]
	MT-ND4, MT-ND5	Esophagus	ND	ND	[81]
	MT-ND5	Melanoma	ND	metabolic reprogramming; immune system evasion	[82]
	MT-ND5	Glioma	↓ complex activity	ND	[83]
	MT-ND1, MT-ND2, MT-ND4, MT-ND5, MT-ND6	Prostata	ND	ND	[84]
	MT-ND2	Fibroblast	↓ respiratory capacity	↑ metastatic ability ↑ tumorigenic potential	[85]
**Complex II**	SDHA, SDHB, SDHC, SDHD, SDHAF2	Hereditary paraganglioma/phaeochromocytoma syndrome	↓ complex activity	↑ succinate (oncometabolite)	[86,87]
	SDHA, SDHB, SDHC, SDHD	Gastrointestinal	↓ complex activity	↑ succinate (oncometabolite) ↑ therapy resistance	[88]
	SDHA, SDHB, SDHC, SDHD	Renal carcinoma	↓ complex activity	↑ succinate (oncometabolite)	[89,90]
**Complex III**	MT-CYB	Esophagus	ND	ND	[81]
	MT-CYB	Colon	ND	ND	[65,91]
	MT-CYB	Pancreas	ND	ND	[69,70]
	MT-CYB	Ovarian	ND	ND	[92]
	MT-CYB	Prostata	ND	ND	[84]
	MT-CYB	Bladder	↓ complex activity ↑ ROS production	↑ metastatic ability ↑ proliferation ↓ apoptosis	[93,94]
	MT-CYB	Oral cavity	ND	ND	[95]
	MT-CYB	Glioma	ND	ND	[96]
	UQCRC1	Mesotelioma	ND	↓ survival	[97]
**Complex IV**	MT-CO3	Leukemia	↓ complex activity	ND	[79]
	MT-CO1, MT-CO2	Lung [64,65,69,70,91]	ND	ND	[77]
	MT-CO2, MT-CO1, MT-CO3	Colon	ND	ND	[64,65,91]
	MT-CO1, MT-CO2, MT-CO3	Pancreas	ND	ND	[69,70]
	MT-CO1, MT-CO2, MT-CO3	Prostate	ND	ND	[84,98]
	MT-CO1, MT-CO2, MT-CO3, CO4I2, COX5A, COX5B, COX6A2, COX6C, COX7B2	Breast	ND	ND	[72,99]
**Complex V**	MT-ATP6, MT-ATP8	Leukemia	ND	ND	[79]
	MT-ATP6	Lung	ND	ND	[76]
	MT-ATP6	Pancreas	ND	ND	[69,70]
	MT-ATP6	Breast	ND	ND	[73]
	MT-ATP6	Prostate	↑ ROS production	↑ proliferation	[84,98]
	MT-ATP6	Glioma	ND	ND	[64]

MT-ND1-6: mitochondrially encoded NADH–ubiquinone oxidoreductase core subunit 1-6; MT-ND4L: mitochondrially encoded NADH–ubiquinone oxidoreductase core subunit 4L; SDHA: succinate dehydrogenase complex flavoprotein subunit A; SDHB: succinate dehydrogenase complex iron sulfur subunit B; SDHC-D: succinate dehydrogenase complex subunit C-D; SDHAF2: succinate dehydrogenase complex assembly factor 2; MT-CYB: mitochondrially encoded cytochrome b; UQCRC1: ubiquinol–cytochrome c reductase core protein 1; MT-CO1-3: mitochondrially encoded cytochrome c oxidase I–III; COX4I2: cytochrome c oxidase subunit 4I2; COX5A-B: cytochrome c oxidase subunit 5A–B; COX6A2: cytochrome c oxidase subunit 6A2; COX6C: cytochrome c oxidase subunit 6C; COX7B2: cytochrome c oxidase subunit 7B2; MT-ATP6: mitochondrially encoded ATP synthase membrane subunit 6; MT-ATP8: mitochondrially encoded ATP synthase membrane subunit 8.

**Table 2:** Mitochondrial antioxidant systems.

Antioxidant	Mechanism	Cancer Type	Expression	Tumor Activity	Reference
SOD	Catalyze dismutation of O_2_•^−^ into H_2_O_2_ [165,166,167,168].	Colon, lung, prostate	↑	↑ metastasic potential	[169]
		Prostate	↓	ND	[170]
		Pancreatic	↑	↓ tumor growth	[171]
		Breast	↑	↓ ROS	[114]
		Lung	↑	Maintains oncogenic capacity ↑ tumorigenesis	[116,172]
		Prostate	↑	ND	[118]
Catalase	Breaks down H_2_O_2_ into O_2_ and H_2_O [173,174,175,176,177].	Glioma	↑	↑ Treatment resistance	[178]
		Breast	↑	↑ invasiveness ↑ metastasic potential	[179]
Thiodoredoxin	NADPH + H^+^ reduces oxidized Trx reductase, which regenerates the pool of reduced Trx [180,181,182,183].	Melanoma	↑	↑ metastasic potential	[184]
Glutathione peroxide	Catalyzes the reduction of H_2_O_2_ by GSH [127,185,186].	Breast	↑	ND	[187]
Glutathione	The active thiol group of GSH is easily oxidized and dehydrogenated, thus eliminating O_2_•^-^ and providing e^−^ for enzymes such as GPX to reduce H_2_O_2_ [188,189,190,191,192].	Breast	↑	ND	[187]
Alpha lipoic acid	DHLA (reduced form) of ALA donates e^−^ to a pro-oxidant or an oxidized molecule. It can regenerate ascorbic acid from dehydroascorbic acid, and it can indirectly regenerate vitamin E back from its oxidized state [144,193,194,195,196].	Neuroblastoma Breast	↑	↓ cell viability and proliferation ↑ apoptosis	[149]
		Liver	↑	↓ tumor growth	[197]
		Prostate	↑	↓ cell viability ↓ clonogenic capacity ↑ ROS	[198]
		Lung	↑	↓ cell viability and proliferation	[146]
Coenzyme Q (Ubiquinone)	At the cytosolic side of the plasma membrane, a quinone reductase reduces CoQ to ubiquinol (CoQH2). CoQH2 shuttles e^−^ to the NOX, leading to reduced extracellular ascorbyl radicals, reduced O_2_ to water, and reduced protein disulfides [199,200,201]	Breast	↓	ND	[156,202]
		Breast	↑	ND	[203]
NADPH	Serves as the final donor of reducing power for the majority of ROS detoxifying enzymes [161,204,205,206]	Hepatocarcinoma	↑	Improves oxidative stress tolerance, facilitates survival in hypoxic environments	[207]
		Pancreas	↓	↓ clonogenic capacity	[171]
		Gastric	↑	↓ tumor angiogenesis	[208]
		Melanoma	↓	Promotes tumor differentiation ↓ proliferation	[209]

**Table 3:** Lipid composition of rat liver outer and inner mitochondrial membranes.

A.	OMM	IMM
Phospholipids (mg/mg protein)	0.45	0.20
Cholesterol (mg/mg protein)	0.04	<0.01
**B.**	**% of Total Phospholipids**	
Phosphatidylcholine	54	40
Phosphatidylethanolamine	29	34
Cardiolipin	<1	18
Phosphatidylinositol	13	5
Phosphatidylserine	2	3
Phosphatidic acid	1	-

Data from [306]. (A) Content of total phospholipid and cholesterol rat liver mitochondria. (B) Phospholipid classes. Note that cardiolipin is mostly concentrated in the IMM. IMM: inner mitochondrial membrane; OMM: outer mitochondrial membrane.

## Data Availability

Data are contained within the article.
